# Current evidence of neurological features, diagnosis, and neuropathogenesis associated with COVID-19

**DOI:** 10.1590/0037-8682-0534-2021

**Published:** 2022-02-25

**Authors:** Marzia Puccioni-Sohler, André Rodrigues Poton, Milena Franklin, Samya Jezine da Silva, Rodrigo Brindeiro, Amilcar Tanuri

**Affiliations:** 1 Universidade Federal do Estado do Rio de Janeiro, Escola de Medicina e Cirurgia, Rio de Janeiro, RJ, Brasil.; 2 Universidade Federal do Rio de Janeiro, Programa de Pós-Graduação em Doenças Infecciosas e Parasitárias, Rio de Janeiro, RJ, Brasil.; 3 Universidade Federal do Rio de Janeiro, Instituto de Biologia, Rio de Janeiro, RJ, Brasil.

**Dear Editor**

We thank Dr. Josef Finsterer for the letter entitled “Pathophysiological aspects of neuro-COVID. Short title: Pathophysiology of neuro-COVID” and the interest in our publication^1^. The letter highlights how the neurological complications associated with COVID-19 can be widespread^1^. Dr. Finsterer cites three other pathophysiological mechanisms that can cause secondary neurological complications such as cardiac involvement, intensive care unit treatment, and neuro- or myotoxicity of anti-COVID-19 drugs^2^ ^-^ ^7^. Although most of the references cited by the author were conducted prior to the COVID-19 pandemic and based on case reports, numerous other clinical conditions that can cause secondary damage to the nervous system exist. In addition to the letter, there are reports of acute kidney disease, septic shock, and liver and pancreatic dysfunction, among others^8^ ^-^ ^10^. Post-acute COVID-19 syndrome including cognitive decline has also been reported^11^ ^,^ ^12^. Neurotoxicity or myotoxicity of drugs used in the treatment of COVID-19 can occur in any other disease treated by any drug. Thus, these are drug reactions and not COVID-19 mechanisms.

Our study aimed to introduce an issue that emerged within 7 months of the COVID-19 pandemic in 2020, the mechanisms of nervous system infection, and the absence of the virus in the cerebrospinal fluid^1^. These topics have widely been discussed in medical literature. We also highlighted the alarming epidemiological, clinical, and neurological findings reported for COVID-19 and the limitations in the laboratory diagnosis of neuro-COVID-19, considering the frequent negative SARS-CoV-2 real-time reverse transcription polymerase chain reaction test result in the cerebrospinal fluid.

In conclusion, although our study was mainly focused on the emergence of a new neuroinvasive virus and its implications for neurological diagnosis, we agree that a broad study that would generate data on adverse reactions of drugs used in COVID-19 therapy as well as other secondary neurological complications would be of great importance.
